# Antinociceptive effect of methanol extract of leaves of *Persicaria hydropiper* in mice

**DOI:** 10.1186/s12906-015-0558-y

**Published:** 2015-03-13

**Authors:** Ambia Khatun, Mohammad Zafar Imam, Md Sohel Rana

**Affiliations:** Department of Pharmacy, Stamford University Bangladesh, 51 Siddeswari Road, 1217 Dhaka, Bangladesh; Department of Pharmacy, Jahangirnagar University, 1342 Dhaka, Savar Bangladesh

**Keywords:** *Persicaria hydropiper*, Polygonaceae, Antinociceptive, Pain, Opoid system

## Abstract

**Background:**

*Persicaria hydropiper* (Linn.) Delarbre is a common plant of Polygonaceae family commonly called Bishkatali in Bangladesh. Leaves of the plant are traditionally used in the treatment of rheumatic pain, gout, and skin diseases such as ringworms, scabies, boils, abscesses, carbuncles, bites of snakes, dogs or insects. This study evaluated the antinociceptive effect of the methanol extract of *P. hydropiper* leaves (MEPH).

**Methods:**

The antinociceptive activity of MEPH was investigated using heat-induced (hot-plate and tail-immersion test) and chemical-induced (acetic acid, formalin, glutamic acid, cinnamaldehyde) nociception models in mice at 25, 50, and 75 mg/kg doses. Involvement of opioid system, cyclic guanosine monophosphate (cGMP) pathway, and ATP-sensitive K^+^ channel pathway were also tested using naloxone, methylene blue and glibenclamide respectively.

**Results:**

MEPH showed antinociceptive activity in both heat- and chemical induced pain models. In both hot plate and tail immersion tests MEPH significantly increases the latency to the thermal stimuli. In acetic acid-induced writhing test the extract inhibited the number of abdominal writhing. Likewise, MEPH produced significant dose-dependent inhibition of paw licking in both neurogenic and inflammatory pain induced by intraplantar injection of formalin. Besides, MEPH also significantly inhibited the glutamate-induced pain and cinnamaldehyde-induced pain in mice. It was also clear that pretreatment with naloxone significantly reversed the antinociception produced by MEPH in hot plate and tail immersion test suggesting the involvement of opioid system in its effect. In addition, administration of methylene blue, a non specific inhibitor of NO/guanylyl cyclase, enhanced MEPH induced antinociception while glibenclamide, an ATP-sensitive K^+^ channel antagonist, could not reverse antinociceptive activity induced by MEPH.

**Conclusion:**

Based on the results of the current study it can be said that MEPH possesses significant antinociceptive activity which acts in both peripheral and central mechanisms.

## Background

*Persicaria hydropiper* (Linn.) Delarbre is a common plant of Polygonaceae family locally known as Bishkatali in Bangladesh. *Persicaria*, a clade of Polygonaceae containing approximately 120 species, grows at the bank of rivers, canals, lakes and roadsides of tropical and sub-tropical countries [1]. In the traditional medicine leaves are used to treat rheumatic pain, knee pain, gout, skin diseases such as ringworms, scabies, boils, abscesses, carbuncles, bites of snakes, dogs or insects, colic pain, diuretic, and amenorrhea. Sometimes, the leaves are also used as a condiment or flavoring agent [2]. Polygonums are also considered an ideal famine food because of its easy availability and high nutritional values. They are predicted to be an important source to combat nutritional deficiency during the scarcity of food [3-5].

Different types of flavonoids, terpenoids, anthraquinones, apianen lactones and steroids have been isolated from different species of *Persicaria*. A number of bioactive constituents have also been reported from various *Persicaria* species with anticancer, antioxidant, antinociceptive, antileukemic, antimicrobial and tyrosinase-inhibitory activities [6]. The plant has been reported to possess bitter, stimulant, tonic, diuretic, carminative, emmeragogue, haemostatic and lithotripter properties [7]. The plant is also used to treat diarrhoea, dyspepsia, itching skin, excessive menstrual bleeding and hemorrhoids either alone or with other herbs [8].

The use of *P. hydropiper* leaves in different painful conditions in folk medicine and lack of scientific study reporting its antinociceptive activity in different animal models convinced us to evaluate the antinociceptive effect of methanol extract of *P. hydropiper* leaves in different peripheral and central pain models in mice.

## Methods

### Plant material and extract preparation

The leaves of *P. hydropiper* were collected from Katakhali, Bogra, Bangladesh in August, 2012. The collected samples were then identified by Sarder Nasir Uddin, Principal Scientific Officer, Bangladesh National Herbarium, Mirpur, Dhaka, Bangladesh. A voucher specimen has been deposited in the Herbarium for further reference (DACB: 35459). Powdered dried leaves (65.29 g) were macerated with 250 ml of methanol with occasional stirring at 25 ± 2°C for 3 days. The extract was then filtered using a Buchner funnel and a sterilized cotton filter. The solvent was removed by rotary evaporator and 15.95 g extract was obtained (Yield 21.72%). This crude extract was used for the acute toxicity and antinociceptive activity studies.

### Chemicals

The following drugs and chemicals were used in the current study: morphine sulphate, diclofenac sodium, naloxone (Hameln Pharmaceuticals GmbH), acetic acid, methanol, formalin, cinnamaldehyde, methylene blue, L-glutamic acid (Merck, Germany), glibenclemide (Square Pharmaceuticals Ltd., Bangladesh), and DMSO (Merck, Germany).

### Animals

Swiss albino mice (20-25 g) were collected from Animal Resources Branch of the International Center for Diarrhoeal Disease Research, Bangladesh (icddr,b). The animals were kept in standard laboratory conditions (relative humidity 55-60%; room temperature 25 ± 2°C; 12 h light/dark cycle) and were provided with standard diet (icddr,b formulated) and clean water *ad libitum*. The animals were acclimatized to the laboratory environment for a period of 14 days prior to performing the experiments. The animals were fasted overnight before the experiments. All the experimental animals were treated following the Ethical Principles and Guidelines for Scientific Experiments on Animals (1995) formulated by The Swiss Academy of Medical Sciences and the Swiss Academy of Sciences. All experimental protocols were approved by the Ethics Committee of Stamford University Bangladesh (SUB/IAEC/12.04).

### Drugs and treatments

The standard drug morphine sulphate (5 mg/kg) used in hot plate and tail immersion tests and diclofenac sodium (10 mg/kg) in writhing and licking tests were administered intraperitoneally 15 min before the experiments while the animals in control group received vehicle orally (DMSO) at the dose of 10 ml/ kg body weight 30 min before the experiments. MEPH was orally administered to the test animals 30 min before the experiments at the doses of 25, 50, and 75 mg/kg body weight in both the chemical-induced and heat-induced pain models. Naloxone, a non-selective opioid receptor antagonist, was injected intraperitoneally at 2 mg/kg dose 15 min before the administration of morphine sulphate or MEPH (25, 50, and 75 mg/kg) to investigate involvement of opioid receptor system. Methylene blue, a non specific inhibitor of NO/guanylyl cyclase (20 mg/kg) and glibenclamide, an ATP-sensitive K^+^ channel inhibitor (10 mg/kg) were also injected intraperitoneally to test the involvement of cGMP and ATP-sensitive K^+^ channel pathway respectively.

### Acute toxicity test

Swiss albino mice were divided into control and test groups containing five animals each. MEPH was administered orally at the doses of 250, 500, 1000, and 2000 mg/kg. The mice were allowed food and water *ad libitum* and all animals were observed for abnormal behaviors, allergic symptoms and mortality for the next 72 h [9].

### Phytochemical screening

MEPH was qualitatively tested for the detection of carbohydrates, saponins, flavonoids, tannins, alkaloids, glycosides, glucosides, reducing sugars, proteins, gums, and steroids following standard procedures [10].

### Antinociceptive analysis

#### Hot plate test

The mice that showed forepaw licking, withdrawal of the paw(s) or jumping response within 15 s on hot plate kept at a temperature of 50 ± 0.5°C were selected for this study 24 h prior to the experiment. Mice were fasted overnight with water given *ad libitum*. The animals were treated with morphine or MEPH and were placed on Eddy’s hot plate kept at a temperature of 50 ± 0.5°C. A cut off period of 20 s was maintained to avoid paw tissue damage [11]. The response in the form of forepaw licking, withdrawal of the paw(s) or jumping was recorded at 30, 60, 90, and 120 min following treatment.

#### Tail immersion test

To evaluate the central analgesic property the tail immersion test was performed. This procedure is based on the observation that morphine like drugs prolongs the tail withdrawal time from hot water in mice [12]. One to two cm of tail of the mice pretreated with morphine or MEPH were immersed in warm water kept constant at 54 ± 0.5°C. The latency between tail submersion and deflection of tail was recorded. A latency period of 20 s was maintained to avoid tail tissue damage in mice. The latency period of the tail-withdrawal response was taken as the index of antinociception and was determined at 30, 60, 90, and 120 min after the administration of morphine or MEPH.

#### Acetic acid-induced writhing test

Acetic acid-induced writhing test was performed to evaluate the peripheral antinociceptive activity of MEPH in chemical-induced pain. The mice were treated with drug or MEPH and then the writhing was induced by injecting 0.6% acetic acid after 15 and 30 min, respectively, at the dose 10 ml/kg body weight. Five min after the injection of acetic acid, the mice were observed and the number of writhing was counted for 30 min [13]. The contractions of the abdomen, elongation of the body, twisting of the trunk and/or pelvis ending with the extension of the limbs were considered as complete writhing.

### Formalin-induced nociception

Mice were injected with 20 μl of a 2.5% formalin solution (0.92% formaldehyde) made up in saline into the sub-plantar region of the right hind paw 60 min after MEPH treatment and 15 min after injection of diclofenac sodium. Licking of the injected paw was recorded as nociceptive response from 0-5 min (neurogenic phase) and 15-30 min (inflammatory phase) after formalin injection [14,15].

### Glutamate-induced nociception

20 μL of glutamate (10 μmol/paw) was injected into the ventral surface of the right hind paw of mice 30 min after MEPH treatment and 15 min after injection of diclofenac sodium. The mice were observed for 15 min following glutamate injection and number of paw licking was counted as an indication of nociception [16].

### Cinnamaldehyde-induced nociception

20 μl of cinnamaldehyde (10 nmol/paw prepared in saline) were injected intraplantarly in the ventral surface of the right hind paw. Animals were observed individually for 5 min following cinnamaldehyde injection. The amount of time spent licking the injected paw was recorded with a chronometer and was considered as indicative of nociception. The animals were treated with MEPH orally 1 hr before cinnamaldehyde injection. Control animals received vehicle (10 ml/kg orally) [14,17].

### Analysis of the possible mechanism of action of MEPH

#### Involvement of opioid system

The possible participation of the opioid system in the antinociceptive effect of MEPH was examined by injecting naloxone hydrochloride (2 mg/kg i.p.), a non-selective opioid receptor antagonist, 15 min prior to the administration of either morphine or MEPH. Then the hot plate and tail immersion latencies were measured at 30, 60, 90 and 120 min with the same cut off time of 20 s for the safety of animals [18].

#### Involvement of cyclic guanosine monophosphate (cGMP) pathway

To verify the possible involvement of cGMP pathway in the antinociceptive action caused by MEPH the mice were pre-treated with methylene blue (20 mg/kg), a non specific inhibitor of Nitric oxide/guanylyl cyclase, intraperitonially 15 min before administration of either diclofenac sodium or MEPH. Then the nociceptive responses against 0.6% acetic acid injection were observed for 30 min, starting from 5 min post injection. The numbers of abdominal writhing were counted as indication of pain behavior [19,20].

#### Involvement of ATP-sensitive K^+^ channel pathway

Possible contribution of K^+^ channel in the antinociceptive effect of MEPH was evaluated by previously described method [20,21]. The mice were pretreated with glibenclamide (10 mg/kg), an ATP-sensitive K^+^ channel inhibitor, intraperitonially 15 min before administration of either diclofenac Sodium or MEPH. The mice were challenged with i.p. injection of 0.6% acetic acid, 30 min post-treatment. Following the injection of acetic acid, the animals were immediately placed in a Perspex chamber and the number of writhing was recorded for 30 min, starting from 5 min post injection.

### Statistical analysis

The results are presented as MEAN ± SEM. The statistical analysis of the results was performed using one way analysis of variance (ANOVA) followed by Dunnett’s post hoc test or Bonferroni’s test as appropriate using SPSS 11.5 software. Differences between groups were considered significant at a level of p < 0.001 and p < 0.05.

The results of the tail immersion and hot plate tests were given with percentage of the maximal possible effect (% MPE), which was calculated using the following formula.

$$ \%\ \mathrm{M}\mathrm{P}\mathrm{E}=\left[\left(\mathrm{postdrug}\ \mathrm{latency}\right)-\left(\mathrm{predrug}\ \mathrm{latency}\right)/\left(\mathrm{cutoff}\ \mathrm{time}\right)-\left(\mathrm{predrug}\ \mathrm{latency}\right)\right]\times 100 $$

## Results

### Phytochemical screening

The preliminary screening revealed the presence of alkaloid, carbohydrate, glycoside, steroid, flavonoid, saponin and tannin in MEPH.

### Acute toxicity

From the acute toxicity test it has been found that the LD_50_ of MEPH is 758.58 mg/kg in mice.

### Hot plate test

The antinociceptive effect of MEPH and morphine in hot plate test are given in Table 1. MEPH at 50 and 75 mg/kg doses significantly increased the reaction time to the thermal stimulus (p < 0.05). The antinociceptive effect was dose-dependent as we observed stronger effect at 75 mg/kg dose than 50 mg/kg dose. Morphine showed highest latency at all the observation periods. The extract also showed significant increase in latency to the thermal stimuli at 25, 50, and 75 mg/kg doses (p < 0.05). Naloxone exerted significant (p < 0.05) antagonistic effect on the antinociceptive activity of MEPH and morphine.

**Table 1:** **Effect of leaf extract of** ***P. hydropiper*** **extract in hot plate test**

**Treatment**	**Dose ** **(mg/kg)**	**Latency period (in sec) (% MPE)**				
		**Pretreatment**	**30 min**	**60 min**	**90 min**	**120 min**
Control	0.1 ml/mouse	5.97 ± 0.29	6.98 ± 0.32	7.23 ± 0.07	7.88 ± 0.19	9.08 ± 0.21
Morphine	5	6.94 ± 0.62	11.93 ± 0.17*(38.20)	13.34 ± 0.40*(17.47)	15.01 ± 0.08*(25.07)	15.95 ± 0.17* (18.83)
MEPH	25	5.80 ± 0.19	6.86 ± 0.24(11.93)	7.11 ± 0.17(1.90)	7.87 ± 0.20(5.89)	8.08 ± 0.11*(1.73)
MEPH	50	5.98 ± 0.19	6.39 ± 0.10(2.92)	7.80 ± 0.09(10.36)	8.33 ± 0.08(4.35)	10.15 ± 0.05*(15.59)
MEPH	75	6.55 ± 0.82	8.83 ± 0.05*(21.41)	10.26 ± 0.05*(12.80)	12.61 ± 0.08*(24.13)	14.07 ± 0.04*(19.76)
NLX	2	12.79 ± 0.26	10.09 ± 0.06	7.79 ± 0.20	6.85 ± 0.12	5.85 ± 0.19
NLX + Control	2 + 0.1 ml/mouse	5.82 ± 0.29	7.26 ± 0.08	7.66 ± 0.09	8.01 ± 0.08	9.44 ± 0.05
NLX + Morphine	2 + 5	7.29 ± 0.42	7.35 ± 0.58^a^(0.47)	8.21 ± 0.26^a^(6.79)	8.95 ± 0.21^a^ (6.27)	9.13 ± 0.08^a^(1.62)
NLX + MEPH	2 + 25	5.78 ± 0.21	6.07 ± 0.28^b^(2.03)	7.49 ± 0.07(10.23)	8.01 ± 0.13^b^(4.15)	9.09 ± 0.14^b^(9.00)
NLX + MEPH	2 + 50	5.98 ± 0.17	6.35 ± 0.08^c^(2.63)	7.80 ± 0.18(10.62)	8.24 ± 0.08^c^(3.60)	9.23 ± 0.04^c^(8.41)
NLX + MEPH	2 + 75	6.17 ± 0.16	7.05 ± 0.24^d^(6.36)	8.24 ± 0.04(9.18)	8.62 ± 0.06^d^(3.23)	9.43 ± 0.03^d^(7.16)

Each value is presented as the mean ± SEM (n = 5). MEPH = Methanolic extract of *P. hydropiper* leaves; NLX = Naloxone.

*p < 0.001 compared with the control group (Dunnett’s test).

^a^p < 0.001 compared with the morphine group (Bonferroni’s test).

^b^p < 0.05 compared with the MEPH 25 group (Bonferroni’s test).

^c^p < 0.05 compared with the MEPH 50 group (Bonferroni’s test).

^d^p < 0.01 compared with the MEPH 75 group (Bonferroni’s test).

### Tail immersion test

The antinociceptive activity of MEPH and morphine in tail immersion test has been given in Table 2. MEPH at all three doses (25, 50, and 75 mg/kg) significantly increased the latency period to hot-water induced thermal stimuli (p < 0.001) in a dose-dependent manner. Morphine showed highest latency, however, the extract also showed significant latency at 25, 50, and 75 mg/kg doses (p < 0.001) at different observation time. Naloxone exerted significant (p < 0.05) antagonistic effect on the antinociceptive activity of MEPH at all three doses and of morphine throughout the observation periods.

**Table 2:** **Effect of leaf extract of** ***P. hydropiper*** **extract in tail immersion test**

**Treatment**	**Dose (mg/kg)**	**Latency period (in sec) (% MPE)**				
		**Pretreatment**	**30 min**	**60 min**	**90 min**	**120 min**
Control	0.1 ml/mouse	1.93 ± 0.12	2.01 ± 0.13	2.27 ± 0.12	2.31 ± 0.20	2.51 ± 0.17
Morphine	5	2.48 ± 0.28	3.93 ± 0.19*(8.27)	6.13 ± 0.32*(13.69)	9.34 ± 0.11*(23.14)	4.35 ± 0.22(31.88)
MEPH	25	2.63 ± 0.10	5.25 ± 0.09*(15.07)	5.55 ± 0.22* (2.03)	6.22 ± 0.23*(4.63)	4.25 ± 0.10(-14.29)
MEPH	50	2.77 ± 0.33	5.67 ± 0.19*(16.83)	5.87 ± 0.17*(1.39)	6.49 ± 0.21(4.38)	5.36 ± 0.42*(-8.43)
MEPH	75	2.57 ± 0.29	3.31 ± 0.55*(4.24)	3.49 ± 0.23*(1.08)	4.23 ± 0.30*(4.48)	5.08 ± 0.34*(5.38)
NLX	2	4.98 ± 0.09	3.53 ± 0.13	3.68 ± 0.24	3.27 ± 0.18	3.16 ± 0.34
NLX + Control	2 + 0.1 ml/mouse	2.96 ± 0.15	3.14 ± 0.13	3.71 ± 0.24	4.16 ± 0.18	4.11 ± 0.34
NLX+ Morphine	2 + 5	2.59 ± 0.08	2.66 ± 0.21^a^(0.40)	2.72 ± 0.18^a^(0.34)	2.96 ± 0.20^a^(1.39)	2.73 ± 0.18^a^(1.34)
NLX + MEPH	2 + 25	2.35 ± 0.36	2.48 ± 0.17^b^(0.73)	3.31 ± 0.14(4.73)	3.32 ± 0.25^b^(0.05)	3.39 ± 0.16^b^(0.42)
NLX + MEPH	2 + 50	2.43 ± 0.14	2.36 ± 0.19^c^(-0.39)	2.24 ± 0.34^c^(-0.68)	3.19 ± 0.16^c^(5.34)	2.89 ± 0.21^c^(-1.78)
NLX+ MEPH	2 + 75	1.98 ± 0.10	1.95 ± 0.21^d^(-0.16)	1.98 ± 0.17^d^(0.16)	2.65 ± 0.24^d^(3.71)	3.01 ± 0.21^d^(3.63)

Each value is presented as the mean ± SEM (n = 5). MEPH = Methanolic extract of. *P. hydropiper* leaves; NLX = Naloxone.

*p < 0.001 compared with the control group (Dunnett’s test).

^a^p < 0.001 compared with the morphine group (Bonferroni’s test).

^b^p < 0.05 compared with the MEPH 25 group (Bonferroni’s test).

^c^p < 0.01 compared with the MEPH 50 group (Bonferroni’s test).

^d^p < 0.01 compared with the MEPH 75 group (Bonferroni’s test).

### Acetic acid-induced writhing

A significant reduction was observed in the number of writhes induced by acetic acid after MEPH treatment in mice (p < 0.001). It is clear from Table 3 that the antinociceptive activity of MEPH was dose-dependent. The maximum pain relieving effect (67.54%) was given by 75 mg/kg treatment while diclofenac sodium (10 mg/kg) produced a stronger analgesic effect.

**Table 3:** **Effect of leaf extract of** ***P. hydropiper*** **extract in acetic acid-induced writhing test**

**Treatment**	**Dose (mg/kg)**	**Number of writhing**	**Inhibition (%)**
Control	0.1 ml/mouse	64.7 ± 2.96	-
Diclofenac sodium	10	15.6 ± 1.47*	75.89
MEPH	25	48.7 ± 1.92*	24.73
MEPH	50	37.8 ± 1.32*	41.58
MEPH	75	21.0 ± 3.06*	67.54

Each value is presented as the Mean ± SEM (n = 5). MEPH = Methanolic extract of. *P. hydropiper* leaves.

*p < 0.001 compared with the control group (Dunnett’s test).

### Formalin-induced nociception

Oral administration of MEPH at the doses of 25, 50, and 75 mg/kg significantly (p < 0.001) reduced the formalin-induced paw licking in both early and late phases of the test (Table 4). Diclofenac sodium demonstrated complete inhibition of licking in late phase. MEPH has shown a dose-dependent increase in the licking inhibition in both phases.

**Table 4:** **Effect of leaf extract of** ***P. hydropiper*** **extract in formalin-induced nociception**

**Treatment**	**Dose (mg/kg)**	**Licking of the hind paw**			
		**Early phase (0-5 min)**	**Inhibition (%)**	**Late phase (15-30 min)**	**Inhibition (%)**
Control	0.1 ml/mouse	114.6 ± 9.88	-	46.8 ± 5.66	-
Diclofenac sodium	10	22.4 ± 1.83*	80.45	1.4 ± 0.97*	97.00
MEPH	25	69.2 ± 3.61*	39.61	2.4 ± 1.15*	86.75
MEPH	50	46.8 ± 2.69*	59.16	1 ± 0.92*	90.17
MEPH	75	31 ± 3.42*	72.94	2.8 ± 0.86*	94.017

Each value is presented as the Mean ± SEM (n = 5). MEPH = Methanolic extract of *P. hydropiper* leaves.

*p < 0.001 compared with the control group (Dunnett’s test).

### Glutamate-induced nociception

Oral administration of MEPH at 25, 50, and 75 mg/kg doses produced 74.13, 76.92, and 86.71% of inhibition of glutamate-induced nociception respectively. Diclofenac sodium (10 mg/kg) used as standard drug showed 85.31% inhibition compared to the control group. The effect of all the treatment groups was statistically significant (Table 5).

**Table 5:** **Effect of leaf extract of** ***P. hydropiper*** **extract in glutamate-induced nociception**

**Treatment**	**Dose (mg/kg)**	**Licking time (s)**	**Inhibition (%)**
Control	0.1 ml/mouse	159.2 ± 5.74	-
Diclofenac sodium	10	12.20 ± 1.81*	95.10
MEPH	25	41.2 ± 4.92*	74.13
MEPH	50	31.2 ± 6.19*	76.92
MEPH	75	20.8 ± 4.10*	86.71

Each value is presented as the Mean ± SEM (n = 5). MEPH = Methanolic extract of *P. hydropiper* leaves.

*p < 0.001 compared with the control group (Dunnett’s test).

### Cinnamaldehyde-induced nociception

Administration of MEPH at 50, and 75 mg/kg doses produced significant decrease in writhing in cinnamaldehyde-induced nociception with 67.50, and 47.50% inhibition respectively (Table 6). Only 25 mg/kg MEPH treatment showed no significant difference compared with the vehicle group.

**Table 6:** **Effect of leaf extract of** ***P. hydropiper*** **extract on cinnamaldehyde-induced nociception**

**Treatment**	**Dose (mg/kg)**	**Licking time (s)**	**Inhibition (%)**
Control	0.1 ml/mouse	38.6 ± 5.77	-
Diclofenac sodium	10	11.2 ± 4.87*	95.00
MEPH	25	26.6 ± 3.37	2.50
MEPH	50	20.2 ± 3.86*	67.50
MEPH	75	15.2 ± 4.29*	47.50

Each value is presented as the Mean ± SEM (n = 5). MEPH = Methanolic extract of. *P. hydropiper* leaves.

*p < 0.001 compared with the control group (Dunnett’s test).

### Involvement of cyclic guanosine monophosphate (cGMP) pathway

The present study also evaluated the effect of MEPH at 25, 50, and 75 mg/kg doses and methylene blue (20 mg/kg) treatments. MEPH and methylene blue administration alone significantly inhibited acetic acid induced abdominal writhing test. Given together, methylene blue significantly enhanced MEPH induced antinociception compared to the treatment of both MEPH and methylene blue alone (Table 7).

**Table 7:** **Effect of leaf extract of** ***P. hydropiper*** **extract on involvement of cyclic guanosine monophosphate (cGMP) pathway**

**Treatment**	**Dose (mg/kg)**	**Number of writhing**	**Inhibition (%)**
Control	0.1 ml/mouse	59.1 ± 1.86	-
Methylene Blue (MB)	20	52.8 ± 3.15	10.66
MEPH + MB	25 + 20	6.9 ± 0.73*	88.32
MEPH + MB	50 + 20	5.8 ± 1.88*	90.19
MEPH + MB	75 + 20	5.3 ± 3.85*	91.03

Each value is presented as the Mean ± SEM (n = 5). MEPH = Methanolic extract of *P. hydropiper* leaves.

*p < 0.001 compared with the control group (Dunnett’s test).

### Involvement of ATP-sensitive K+ channel pathway

Administration of glibenclamide (10 mg/kg) alone did not alter abdominal writhing after injection of 0.6% acetic acid (Table 8). When glibenclamide was administered with MEPH, the antinociceptive activity of MEPH was not significantly reversed by glibenclamide.

**Table 8:** **Effect of leaf extract of** ***P. hydropiper*** **extract on involvement of ATP-sensitive K** ^**+**^ **channel pathway**

**Treatment**	**Dose (mg/kg)**	**Number of writhing**	**Inhibition (%)**
Control	0.1 ml/mouse	64.7 ± 2.96	-
Glibenclamide (GB)	10	57.4 ± 2.02	11.28
MEPH + GB	25 + 10	29.6 ± 2.31*	54.25
MEPH + GB	50 + 10	25.7 ± 2.59*	60.28
MEPH + GB	75 + 10	22.5 ± 4.33*	65.22

Each value is presented as the Mean ± SEM (n = 5). MEPH = Methanolic extract of *P. hydropiper* leaves.

*p < 0.001 compared with the control group (Dunnett’s test).

## Discussion

The present study demonstrates that oral administration of MEPH elicits a potent and dose-dependent antinociceptive effect in both chemical and thermal-induced nociception models and MEPH acts both centrally and peripherally. The significant increase in latency time in hot plate by MEPH (p < 0.05) at different doses suggests the central antinociceptive activity of MEPH. The effect observed in the tail immersion test, which is selective only for centrally acting analgesics, further supports the outcome of hot plate test [22].

To anticipate the possible mechanism(s) of antinociceptive effect of MEPH we evaluated the effect of naloxone, a non-selective opioid receptor antagonist, against the antinociceptive effect of MEPH. The results of the hot plate and tail immersion test using naloxone indicated that antinociceptive effect of MEPH was reversed by naloxone. The reversal effect was effective as that against morphine in both the hot plate and tail immersion test. These data strongly suggests that the antinociceptive effect of MEPH may occur through opioid receptors at the spinal and supraspinal level. Both tail-immersion and hot-plate tests are based on measuring the response of the animal to thermal stimuli where the tail-immersion monitor a spinal reflex, and the hotplate is used for supraspinal reflex. In agreement with this suggestion, it has been demonstrated that μ2- and δ- opioid receptors are involved in spinal mechanism, while μ1/μ2-opioid receptors may mediate mainly supraspinal analgesia [23]. Therefore, it can be anticipated that the central antinociceptive effect of MEPH may be prominent on μ-opioid receptors.

The acetic acid-induced writhing test has been used for the evaluation of peripheral antinociceptive activity [24]. In addition, writhing response is widely accepted as a model visceral pain due to the release of endogenous mediators of pain, such as prostaglandins, kinins, etc. [25,26]. Oral administration of MEPH produced significant decrease in acetic acid-induced writhing. Intraperitoneal administration of acetic acid causes an increase in the level of cyclooxygenase (COX), lipoxygenase (LOX), prostaglandins (PGs), histamine, serotonin, bradykinin, substance P, IL-1 beta, IL-8, TNF-alpha in the peripheral tissue fluid. Increased level of these mediators causes the excitation of primary afferent nociceptors entering dorsal horn of the central nervous system [27]. The inhibition of writhing response clearly indicates the peripheral antinociceptive effect of MEPH in addition to its central effect. The potent antinociceptive effect of MEPH in the acetic acid-induced model, therefore, suggests that MEPH may be involved in the inhibition of the release or functions of endogenous nociceptive mediators.

In formalin test MEPH showed significant antinociceptive activity in both neurogenic (early phase) and inflammatory (late phase) pain responses. Formalin-induced pain is consistently inhibited by typical analgesic and anti-inflammatory drugs, including diclofenac sodium, morphine, indomethacin and dexamethasone [28,29]. Considering the inhibitory property of MEPH on the second phase of formalin, we might suggest an anti-inflammatory action of the plant extract. Our results showed that MEPH significantly reduced the cinnamaldehyde-induced pain. This result is in line with that obtained in formalin model and indicates that MEPH probably interacts with TRPA1 receptor located in C-fibers reducing the formalin-induced nociception [30].

The oral administration of MEPH also produced a dose-dependent inhibition of the nociceptive response caused by injection of glutamate into the mouse hind paw. It has been reported that this nociceptive response caused by glutamate, involves peripheral, spinal and supraspinal sites of action with glutamate receptors (AMPA, kainate and NMDA receptors), play an important role in modulating this nociceptive response [17,31]. This further suggests that the glutamatergic system is also affected by the PKC (Protein Kinase C). Thus, our results suggest that the antinociception induced by MEPH is associated with its interaction with the glutamatergic system.

Preliminary phytochemical screening has revealed the presence of alkaloids, glycosides, steroids, carbohydrates, saponins, tannins and flavonoids in MEPH*.* Flavonoids have been reported to suppress the intracellular Ca^2+^ level elevation, as well as the release of pro-inflammatory mediators such as TNFα [32]. Tannins have also been reported to contribute in antinociceptive activity [33].

Further experiments were conducted to investigate the participation of cGMP pathway in the antinociceptive effect of MEPH. The activation or deactivation of nociresponsive neurons is dependent on the availability of cGMP. Intracellular cGMP concentrations are regulated by the action of guanylyl cyclases (GCs) and also by the rate of degradation by cGMP-specific phosphodiesterases [34]. Nitric oxide (NO) increases the level of cGMP through the activation of soluble guanylyl cyclase (sGC), which influences a wide range of physiological functions including pain and analgesia [19]. cGMP acts on the ion channels directly or through the activation of protein kinases and phosphodiesterases [35]. In order to prove the involvement/inhibition of cGMP in MEPH -induced antinociception, methylene blue (MB), a guanylyl cyclase and/or nitric oxide synthase (NOS) inhibitor, was administered prior to inducing nociception with i.p. injection of acetic acid. It has been observed that the pretreatment of methylene blue significantly enhanced the antinociceptive effect exerted by MEPH. It has been suggested that MB promotes antinociception by sequentially inhibiting peripheral NOS and GC, resulting in NO interference [19,36].

## Conclusion

In light of the findings of the present study, it can be assumed that the plant extract of *P. hydropiper* possesses remarkable antinociceptive activity. These data lends justifications to the traditional use of the plant in pain and inflammatory disorders. However, further studies are required to understand the underlying mechanism of analgesic activities and to isolate the active principle(s) responsible for the observed pharmacological effects.
